# Defining the substrate envelope of SARS-CoV-2 main protease to predict and avoid drug resistance

**DOI:** 10.1038/s41467-022-31210-w

**Published:** 2022-06-21

**Authors:** Ala M. Shaqra, Sarah N. Zvornicanin, Qiu Yu J. Huang, Gordon J. Lockbaum, Mark Knapp, Laura Tandeske, David T. Bakan, Julia Flynn, Daniel N. A. Bolon, Stephanie Moquin, Dustin Dovala, Nese Kurt Yilmaz, Celia A. Schiffer

**Affiliations:** 1 Department of Biochemistry and Molecular Biotechnology, University of Massachusetts Chan Medical School, Worcester, MA 01605 US; 2 Novartis Institutes for Biomedical Research, Emeryville, CA 94608 USA

**Keywords:** X-ray crystallography, Proteases, Viral infection, Viral proteins

## Abstract

Coronaviruses can evolve and spread rapidly to cause severe disease morbidity and mortality, as exemplified by SARS-CoV-2 variants of the COVID-19 pandemic. Although currently available vaccines remain mostly effective against SARS-CoV-2 variants, additional treatment strategies are needed. Inhibitors that target essential viral enzymes, such as proteases and polymerases, represent key classes of antivirals. However, clinical use of antiviral therapies inevitably leads to emergence of drug resistance. In this study we implemented a strategy to pre-emptively address drug resistance to protease inhibitors targeting the main protease (M^pro^) of SARS-CoV-2, an essential enzyme that promotes viral maturation. We solved nine high-resolution cocrystal structures of SARS-CoV-2 M^pro^ bound to substrate peptides and six structures with cleavage products. These structures enabled us to define the substrate envelope of M^pro^, map the critical recognition elements, and identify evolutionarily vulnerable sites that may be susceptible to resistance mutations that would compromise binding of the newly developed M^pro^ inhibitors. Our results suggest strategies for developing robust inhibitors against SARS-CoV-2 that will retain longer-lasting efficacy against this evolving viral pathogen.

## Abstract (web summary)

The authors determined crystal structures of the main protease (Mpro) of SARS-CoV-2 bound to substrate peptides. These structures define the substrate envelope and enable identification of sites that may be susceptible to drug resistance.

## Introduction

Coronaviruses are a family of non-segmented positive-sense single-stranded RNA viruses, some of which cause human diseases that range in severity from common cold to highly lethal respiratory infections^1–3^. The most recent example of a human pathogen from this family is severe acute respiratory syndrome coronavirus 2 (SARS-CoV-2) that was identified in 2019 as the causative agent of COVID-19. Over the last two years, SARS-CoV-2 has infected more than 380 million people worldwide and caused over 5.7 million deaths according to WHO^4^, resulting in an unprecedented on-going global pandemic. Biomedical research community has responded rapidly to this challenge and strategies have been developed for prevention and treatment of COVID-19, most notably several vaccines that are safe and effective against early SARS-CoV-2 variants^5–7^. However, SARS-CoV-2 has been evolving, with distinct waves of variants, including the most recent highly transmittable omicron variant, which appears to be able to evade antibodies generated by prior infections or vaccination^8–11^. Therefore, development of antiviral therapeutics to treat patients with active SARS-CoV-2 infection remains a priority. In this context, development of drugs that target essential viral enzymes has been of high interest, resulting in recent approval of nirmatlervir, an inhibitor of the SARS-CoV-2 main protease, M^pro^ (also known as 3CL^pro^)^12^.

The SARS-CoV-2 translates its genome into polyproteins that must be cleaved to release individual proteins essential for replication of the virus. Given that M^pro^ processes the majority of these sites, any intervention that stops this process, such as an inhibitor, would block viral growth. Additionally, M^pro^ has also been implicated in cleaving sites in key cellular host factors to likely enhance viral replication^13^, further highlighting the relevance of this enzyme as a drug target. Antiviral drugs that target viral proteases have demonstrated success in HIV-1 and HCV infections^14–17^. Similarly, M^pro^ inhibitors are becoming essential therapeutics for combating SARS-CoV-2 and potential future pandemics.

However, as seen for HIV-1, HCV, and influenza, drug resistance emerges for every antiviral when used individually^18–20^. Even in short lived influenza respiratory infections, the H275Y mutation arose within neuraminidase and was responsible for widespread resistance in 2009 H1N1 to the antiviral oseltamivir^21^. Thus, based on prior experience with viral protease inhibitors^16,22–24^ and influenza neuraminidase inhibitors, resistance to drugs targeting SARS-CoV-2 M^pro^ is likely to emerge. This underscores the need to better understand M^pro^ structure and function, use this information to predict drug resistance mechanisms, and integrate that knowledge into drug design and development process.

In general, the viral proteases bind substrates of diverse amino acid sequences with high specificity, suggesting that substrate recognition is not sequence-based. To explain this molecular recognition, we developed the concept of *substrate envelope*, a conserved three-dimensional structure or shape that defines substrate binding and specificity, and we explored and validated this concept in HIV-1 and HCV NS3/4 A proteases^25–27^. The substrate envelope also explains susceptibility of protease inhibitors to resistance, whereby residues that contact the inhibitor outside the substrate envelope can mutate without affecting substrate recognition, thus conferring resistance^26,28^. In contrast, inhibitors that fit well within the substrate envelope leverage the evolutionary constraint of substrate recognition. Therefore, designing potent protease inhibitors that fit within the framework of substrate envelope would result in inhibitors that are less likely to result in drug resistance.

To define the structural basis of SARS-CoV-2 M^pro^ substrate recognition and thereby determine the envelope to avoid drug resistance, we have determined 9 substrate-cocrystal structures. The high-resolution structures revealed the intermolecular interactions essential for molecular recognition and enabled defining the conserved substrate envelope. Six additional cocrystal structures of M^pro^ bound to the product (cleaved N-terminal side of the substrate peptide) were also determined and comparatively analyzed. Our analysis revealed the intermolecular interactions that are essential for enzymatic function and substrate specificity of M^pro^, as well as suggesting specific residues that are vulnerable to the occurrence of resistance. Therefore, our results provide critical information that will guide the design of M^pro^ inhibitors.

## Results

### Structures of SARS-CoV-2 M^pro^ with substrates and products

**Fig. 1:**
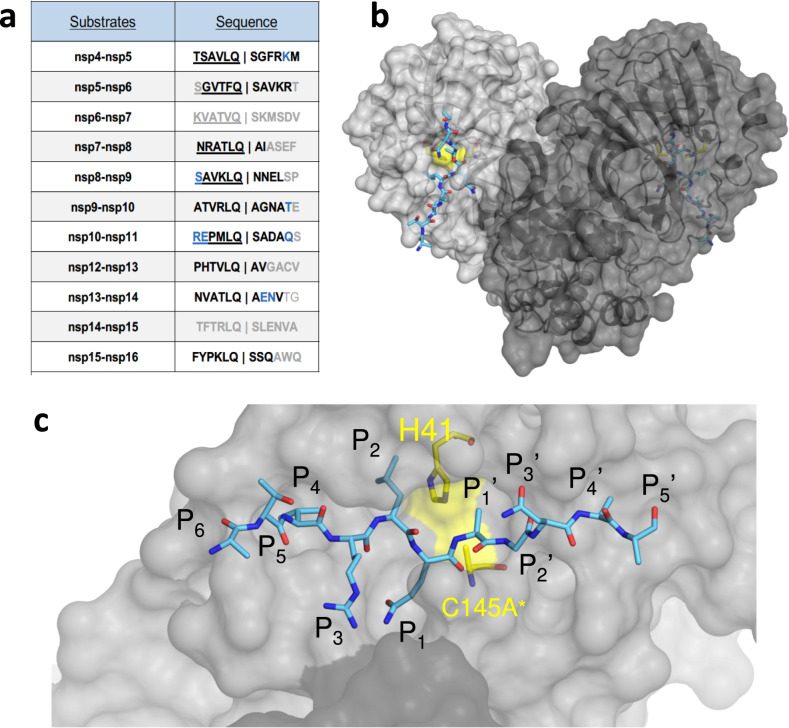
The amino acid sequences and binding of substrates to SARS-CoV-2 M^pro^ active site. **a** Viral polyprotein cleavage sites processed by M^pro^ to release non-structural proteins (nsp). The one-letter amino acid codes of cleavage site sequences, where bold letters indicate fully resolved residues and blue are stubbed side chains in the cocrystal structures. Underlined N-terminal sequences correspond to product complexes with independently determined cocrystal structures. **b** Crystal structure of SARS-CoV-2 M^pro^ with a substrate peptide (nsp9-nsp10) bound at the active site of both monomers (light and darker gray). The peptide is depicted as cyan sticks and the catalytic dyad is colored yellow. **c** Close-up view of one of the active sites in panel B, with the protease in surface representation. The asterisk indicates catalytic cysteine was mutated to prevent substrate cleavage. The cleavage occurs between positions P1 and P1’.

The crystal structures of inactive SARS-CoV-2 M^pro^, with the catalytic Cys145 mutated to Ala, with 9 substrate and 6 product complexes were determined to sub-2.5 Å resolution (Supplementary Tables 1 and 2). There is little sequence conservation among the natural cleavage sequences (Fig. 1a), except the fully conserved glutamine at the P1 position and a hydrophobic residue (Leu/Phe/Val) at the P2 position. We used 12-mer peptides, P6-P6’ (with the scissile bond between P1 and P1'), in our co-crystal structures. The peptides were designed to include the cleavage site, and extend sufficiently to fill the entire substrate binding region. Additionally, from past studies we inferred that although M^pro^ cleaves these sites in the context of a longer polypeptide to release viral non-structural proteins (nsps), molecular interactions with the protease are primarily localized to residues proximal to the scissile bond. Therefore, these cocrystal structures represent a relevant model to understand substrate recognition and specificity of M^pro^. From our cocrystal structures we observed that the peptides were largely ordered from P5-P2’ positions. As in previous apo and inhibitor-bound crystal structures^29–32^, M^pro^ crystallized as a homodimer (Fig. 1b). Six of the nine complexes were solved with the dimer in the asymmetric unit (in P2_1_ or P2_1_2_1_2_1_), with both active sites in the homodimer occupied with the substrate, while the other three had a monomer in the asymmetric unit (in C2_1_). As was previously observed, the N terminal serine residue of one monomer was reaching into the active site of the other monomer, completing the S1 pocket of the other monomer’s active site (Fig. 1c). In these cocrystal structures, both active sites were fully occupied with the noncleaved substrate in essentially the same conformation. This is in contrast to the “half-site” activity previously suggested for SARS-CoV-1 M^pro^^33^, which was recently also proposed for SARS-CoV-2 based on an inactive conformation of Gln166 observed in one of the monomers in an inhibitor-bound structure^31^. In all our structures, Gln166 is in the same “active” conformation in both monomers, thus suggesting that both monomers can be simultaneously active.

We observed that the substrate peptide was extended along the M^pro^ active site with the scissile bond (P1 glutamine to P1’ residue) positioned between the catalytic dyad in all cocrystal structures (Supplementary Fig. 1). The N-terminal (or non-prime) side of the substrate had an antiparallel beta-strand conformation which was conserved in all structures, with the substrate residues and side chains well resolved. The binding mode of the C-terminal residues (prime side) was more varied, especially beyond P3’ position, and lacked full electron density. The N-terminal products (Supplementary Fig. 2) carboxyl terminus is coordinated by the catalytic dyad. In addition to the fully conserved P1 glutamine, which is stabilized through multiple molecular interactions, the large hydrophobic residue at P2 was extended deep into the S2 binding pocket in all structures.

### Precise hydrogen bonding network ensures M^pro^ substrate specificity

**Fig. 2:**
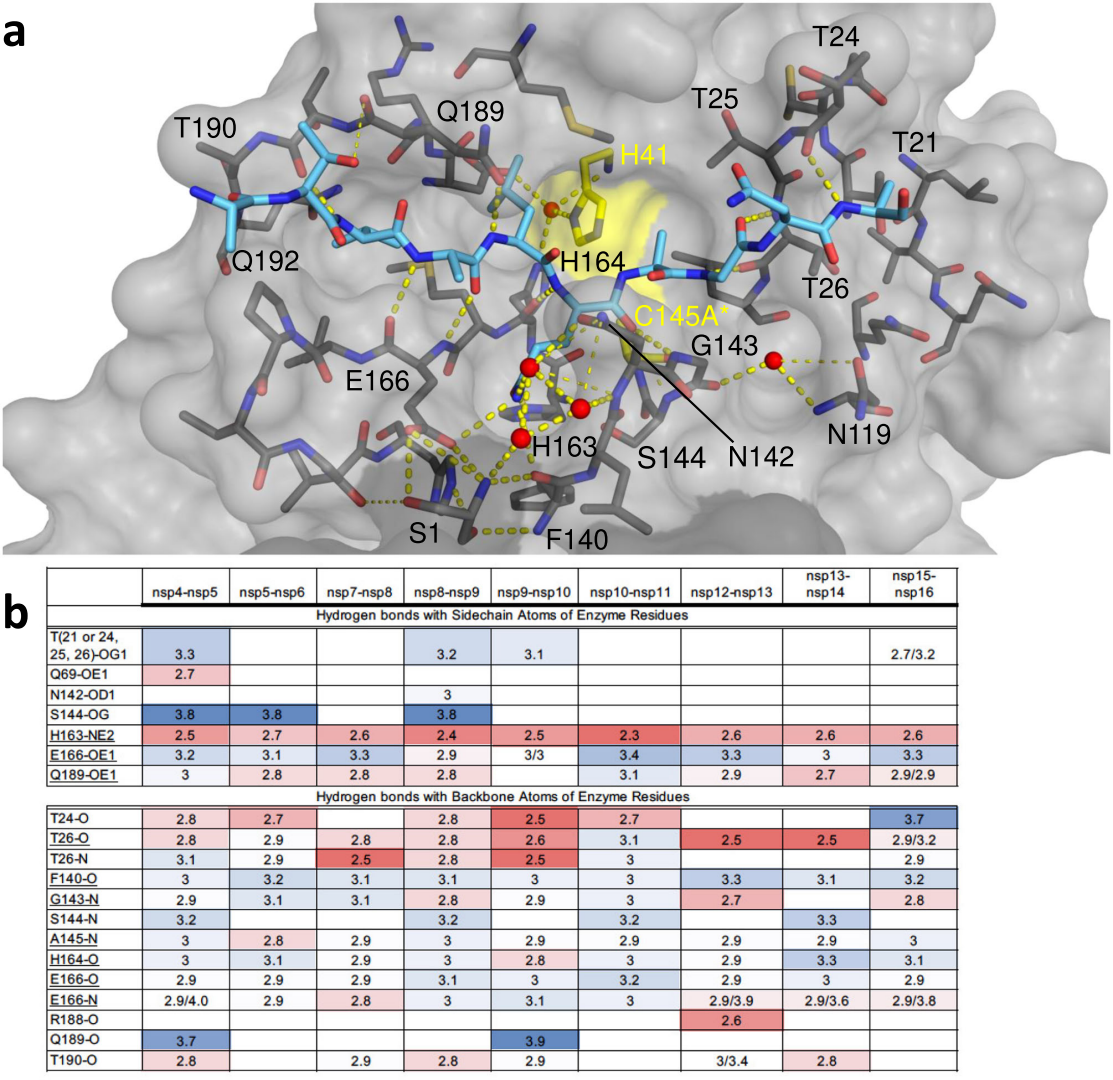
Intermolecular hydrogen bonds in M^pro^ substrate cocrystal structures. **a** Hydrogen bonds between bound nsp9-nsp10 substrate and M^pro^. The substrate peptide is depicted as cyan sticks and the protease is in gray surface representation with the catalytic dyad colored yellow. Yellow dashed lines indicate hydrogen bonds (thicker lines for stronger bonds with distance less than 3.5 Å) and red spheres denote conserved water molecules. Ser1 depicted as sticks belongs to the other monomer (shown in darker gray). **b** Hydrogen bonds that are conserved in three or more substrate complexes; underlined completely conserved, top interacting with M^pro^ sidechains and bottom with M^pro^ backbone atoms, color coded by the distance of the hydrogen bond.

To investigate interactions that stabilize the bound substrate and identify conserved features, we analyzed the intermolecular interactions between the substrates and M^pro^ active site residues. The substrate peptides and the active site residues formed networks of conserved hydrogen bonds that stabilized the binding interaction (Fig. 2) including some mediated by conserved waters. In all of the cocrystal structures, the catalytic His41 was stabilized by a network where a conserved, potentially catalytic water, is coordinated by Asp187 and His164. The sidechain of the conserved P1 glutamine was also extensively coordinated in another hydrogen bonding network. The first shell of this network includes the sidechains of His163 and Glu166, the backbone of Phe140, and three conserved waters. This network was further stabilized by the sidechain and backbone of Asn142 which coordinates the conserved waters and Ser1 from the other monomer, stabilizing the position of both the backbone of Phe140 and the sidechain of Glu166. This extensive network underlies the requirement of homodimer formation in defining the P1 glutamine specificity.

Beyond the P1 sidechain, M^pro^ also established conserved hydrogen bonds with the backbone of the substrates, largely in the form of backbone-backbone hydrogen bonds. The N-terminal side of the P1O was coordinated by three nitrogen atoms (Gly143N, Ser144N and Cys(Ala*)145 N), and P1N also established several conserved intermolecular hydrogen bonds (Fig. 2b). On the C-terminal (prime) side of the substrates, the interactions were again backbone-backbone hydrogen bonds. Each cleavage site was further stabilized by additional sequence-specific hydrogen bonds, often coordinating highly ordered water molecules.

### Packing of diverse substrates is largely conserved with M^Pro^

**Fig. 3:**
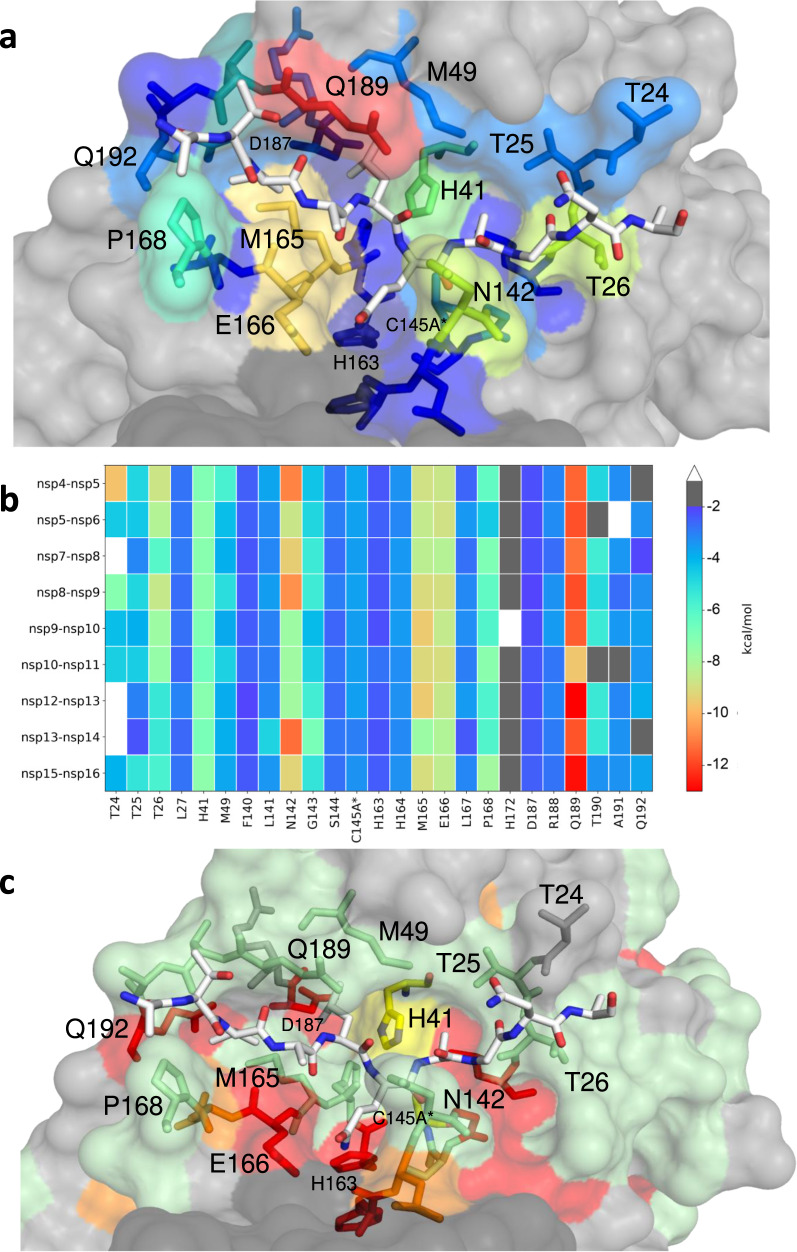
Extent of substrate interactions and conservation of M^pro^ surface residues. **a** Close-up view of the nsp9-nsp10 substrate bound to M^pro^ active site in the cocrystal structure where the substrate peptide is depicted as white sticks and the protease is in surface representation. The protease residues are colored according to the extent of van der Waals interactions with the substrate, with warmer colors indicating more interaction. **b** Conservation of substrate-protease van der Waals interactions among the 9 cocrystal structures determined. Heat map coloring by extent of van der Waals contact by residue. **c** Amino acid sequence conservation of M^pro^ between 7 (Supplementary Fig. 4) coronaviral species depicted on the structure where surface residues conserved in all 7 (red), 5-6 (orange), 3-4 (green) and less than 3 (highly variable; gray) sequences are indicated by color.

To analyze packing of active site residues around the substrate peptides and quantify the inter-molecular interactions, van der Waals (vdW) contacts were calculated for each protease-substrate pair (Fig. 3a). Overall, the contact pattern was consistent among the substrates, where M^pro^ residues at the S3-S2’ sites contributed significant vdW contacts with the substrate (Fig. 3b). At the S1 subsite, the conserved P1 glutamine made substantial vdW interactions with Asn142, consistent with the extensive hydrogen bonding network described above regarding P1 specificity. Significantly, we observed that the sidechain of Gln189 forms a cavity which engulfs the P2 residue and forms the most extensive vdW contacts for each substrate. Met165, Leu167 and Gln192 also form a pocket that accommodates the P4 residue. As prime side residues are poorly conserved between M^pro^ substrates, we identified only a few conserved vdW contacts including the threonine cluster (Thr24, Thr25, and Thr26), which forms hydrogen bonds to stabilize prime side residues prior to substrate cleavage. While not the most extensive, the vdW contacts of catalytic dyad His41 and Cys145A* were highly conserved and consistent between all nine substrates. Analyzing the packing of the substrates, the conserved Gln P1 interacts the most extensively with the enzyme, followed by P2 and P4, and then P1’ (Supplementary Fig. 3). Overall, despite the vast variation in substrate amino acid sequences, the packing around the bound substrates and interactions of protease residues were highly structurally conserved.

Interestingly, coronavirus M^pro^ is not very well-conserved, including around the active site, indicating that most of the residues that form the substrate binding surface tolerate quite a bit of variability (Fig. 3c and Supplementary Fig. 4), which is consistent with our recent mutational analysis^34^. These include Asn142, Met165, Glu166 and Gln189 which form extensive vdW interactions and hydrogen bonds with the substrates. The mutational analysis in the SARS-CoV-2 M^pro^ background revealed that all four residues tolerate extensive variability. While the variability at Asn142, Met165 and Gln189 is in good agreement with sequence variation among coronavirus species (Supplementary Fig. 4), Glu166, which coordinates P1, is conserved. We noticed, very few key conserved residues are within the substrate binding site that are also invariant in the mutational analysis^34^, and we found they play critical structural roles. For instance, invariant His163 forms a completely conserved hydrogen bond with the P1 glutamine (Fig. 2b) and invariant Leu27 lines the P1’ pocket. While the variable Gln189 is located in the center of the structurally and sequence-wise variable loop, this residue is anchored by two completely invariant residues Asp187 and Gln192. Taken together the surface of M^pro^ can tolerate extensive variation while maintaining activity and structural interactions.

### The M^pro^ substrate envelope

**Fig. 4:**
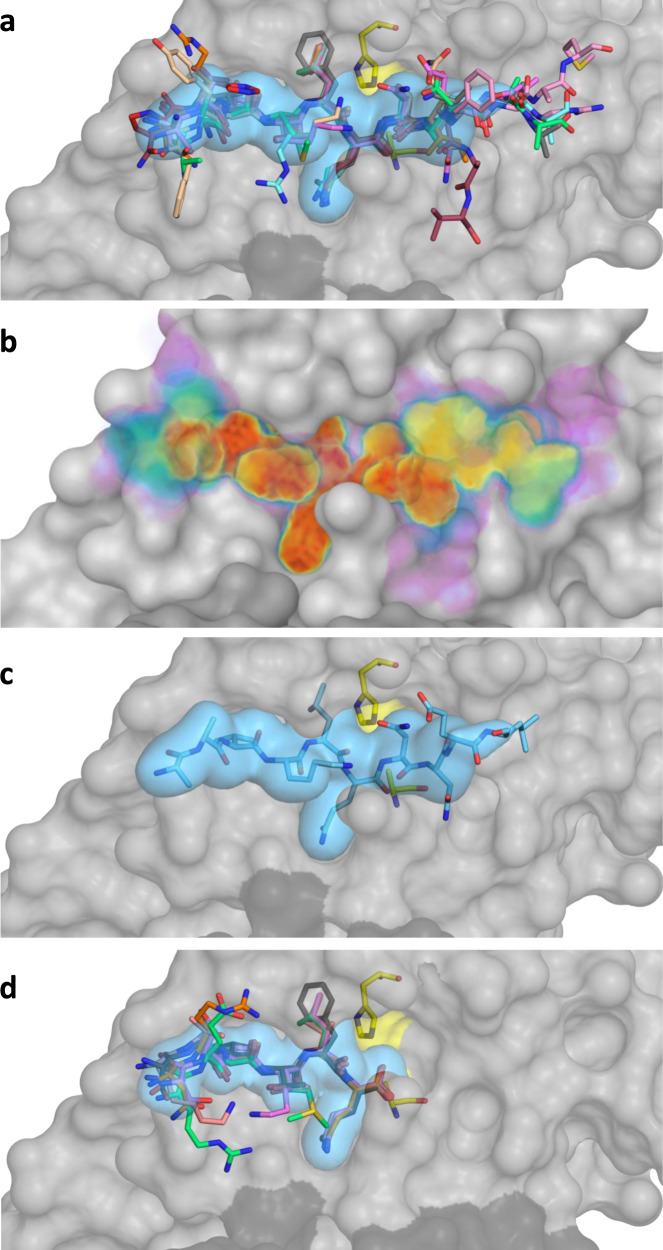
The substrate envelope of SARS-CoV-2 M^pro^. **a** The substrates bound at the M^pro^ active site are depicted as sticks in the superimposed cocrystal structures, where the protease is in gray surface representation. The consensus volume occupied by the substrates define the substrate envelope, shown as the blue volume and is the intersection of any four of the nine substrates. **b** The gradient substrate envelope colored according to the number of substrates that occupy the consensus volume. Purple to red gradient indicates less to more consensus. **c** Substrate nsp8-nsp9 in the substrate envelope. **d** Superposition of cocrystal structures with cleaved N-terminal product complexes, defining the product envelope (blue volume).

The broad range of sequences that M^pro^ cleaves present a challenge to efforts to define key elements of molecular recognition that govern substrate specificity and enzymatic activity. To elucidate how this is achieved, we superimposed our cocrystal structures of substrate-bound M^pro^ based on a set of invariant active site residues (see Methods). The substrate structures superimpose very well, especially the P2-P1’ residues (Fig. 4). Structurally, the M^pro^ enzyme in complex with nsp5-nsp6 displayed the most divergence from the other complexes, likely due to the need to accommodate a unique Phe at P2 rather than the P2 Leu the other substrate sites contain. The divergence was especially in the loop that closes over the substrates (Asp187 to Gln192) (Supplementary Fig. 5), and adaptability of this loop appears to be key to accommodating diverse substrate sequences.

On the molecular level, what accounts for the substrate specificity of M^pro^ with such diverse sequences? As we have previously seen with HIV-1 and HCV NS3/4 A proteases where substrate shape accounts for specificity, despite differences in sequence^25,35^, M^pro^ appears also to recognize a conserved substrate shape. This shape defines the substrate envelope. The substrate envelope was calculated by overlapping consensus volumes to visualize the space occupied at the M^pro^ active site (Fig. 4a). While certain regions, particularly at the C-terminal (prime) side, deviate from the consensus these moieties are largely solvent exposed. To comprehensively evaluate the consensus and conservation of the occupied volume at the M^pro^ active site, we also calculated a gradient substrate envelope reflecting how many substrates overlap at a given position (Fig. 4b). In this gradient envelope, the space occupied by only a single substrate has the lowest score (shown in purple) while the space that all substrates occupy has the highest score (shown in red). As an example, the nps8-nsp9 substrate, which is the most conserved cleavage site between coronaviral species^36^, fits extremely well within the substrate envelope (Fig. 4c). The volume from P4 to P2’ is highly conserved between all of the substrate complexes, despite the variation in amino acid sequences; this high conservation reflects the specificity and likely evolutionarily-constrained regions of the enzyme.

In addition to the full peptide substrates corresponding to the viral polyprotein cleavage sites, we determined structures of six product complexes. The proposed reaction mechanism for M^pro^ involves breakage of the scissile bond and formation of an acyl-enzyme complex with a covalent bond between the N-terminal fragment and catalytic cysteine. Our cocrystal structures captured the N-terminal product after the cleavage reaction was complete where no covalent bond exists with the catalytic cysteine. All 6 cleaved substrates bound at the N-terminal side of the active site superimposed very well, defining a “product envelope” (Fig. 4d). There were no major rearrangements or shifts in the backbone and except minor side chain conformers, the products bound similarly to the noncleaved substrates. The nsp5-nsp6 cleavage site with the P2 Phe was once again the outlier (Supplementary Fig. 5). Overall, the product envelope recapitulated the consensus volume revealed by the substrate envelope for the N-terminal part of the M^pro^ active site.

### Inhibitor fit within the substrate envelope and potential for emergence of resistance mutations

**Fig. 5:**
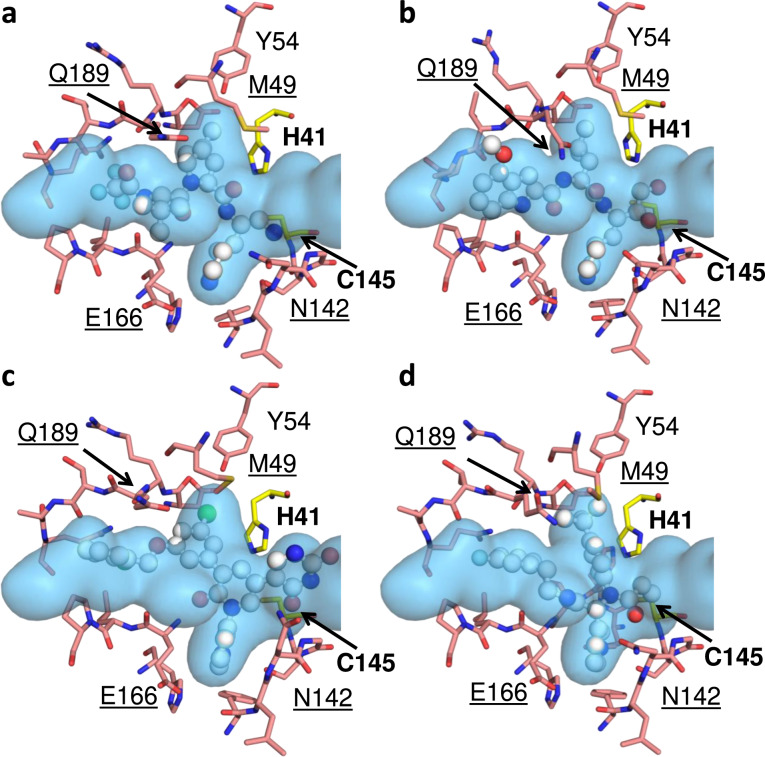
The fit of protease inhibitors within the substrate envelope. **a** PF-07321332 (35), PDB ID:7RFS, **b** PF-00835231 (36), PDB ID 6XHM. **c** Noncovalent potent compound 21 (37), PDB ID: 7L13. **d** Moonshot compound 11 (38), PDB ID 7NW2. The inhibitors are in ball-and-stick representation and the substrate envelope is depicted as the blue volume. The catalytic dyad residues are labeled in bold while the underlined labels are for the residues that interact with one or more inhibitor and may be vulnerable to resistance.

Over the years, we and others have shown that regions where inhibitors protrude from the substrate envelope are susceptible to resistance mutations^16,26,28,37–40^. Thus, our newly determined substrate envelope of M^pro^ provides a predictive tool to assess likelihood that resistance to a specific inhibitor will emerge. To investigate this further, we analyzed binding mode of four SARS-CoV-2 M^pro^ inhibitors that are either under emergency use authorization by the FDA or currently in development: PF-07321332^41^ (Fig. 5a); PF-00835231^42^ (Fig. 5b); a non-covalent inhibitor compound 21^43^ (Fig. 5c); and the most potent covalent inhibitor from the COVID Moonshot project, compound 11^44^ (Fig. 5d). All covalent inhibitors span P4-P1 portion of the active site, while the non-covalent inhibitor, compound 21^43^, extends into the P1’ region. The variation in contact of the inhibitors with the active site can be seen in the variation in vdW contact where Met165 and Glu166 make the most extensive interactions (Supplementary Fig. 6). These four inhibitors all have similar vulnerabilities as assessed by the substrate envelope; the lactam ring and other ringed moieties protrude out at P1 near Asn142 and Glu166 while at P2 inhibitors protrude along the conformationally variable loop from 187-192 and Gln189, and P3 makes extensive contact at Met165. Four residues (Asn142, Gln189, Met49 and Met165) appear very flexible, adopting varied conformations depending on the inhibitor, which is in contrast with their invariant conformations when bound to substrates. These variations in conformation often occur near where the inhibitors protrude from the M^pro^ substrate envelope. These sites are not conserved among coronaviral species and can tolerate mutations^34^, rendering these residues potentially vulnerable to emergence of drug resistance mutations. Additionally, although the conformation of Glu166 is conserved structurally, this residue also appears to tolerate mutations^34^, suggesting that it needs to be taken into account when designing inhibitors to M^pro^. Overall, this analysis suggests that SARS-CoV-2 has the capacity to evolve resistance to the four compounds we tested through changes in residues 49, 142, 165, 166 and 189. We predict that the virus could achieve this either by mutating current sidechains with more rigid residues (His, Tyr, Trp or Phe), thus causing a clash, or by introducing smaller residues (Ala, Thr, Val, Leu, Ser) to cause a loss of contact. These changes could dramatically alter drug binding, while maintaining the substrate envelope and recognition.

## Discussion

The urgency of the COVID-19 pandemic resulted in the fastest development of antiviral drugs to date. However, while this is a breakthrough accomplishment, the emergence of new SARS-CoV-2 variants and the possibility of future pandemics arising from natural zoonotic coronavirus reservoirs present a challenge to developing an arsenal of drugs with durable efficacy. Therefore, taking preemptive strategies to design inhibitors that are less susceptible to drug resistance are essential. Here, we describe our efforts to address this challenge by analyzing substrate binding specificity and recognition of SARS-CoV-2 M^pro^, a viral protease that has garnered a lot of attention as a drug target for treatment of COVID-19. Initial efforts by both academia and industry have successfully identified a variety of M^pro^ inhibitors through a combination of structure-based and medicinal chemistry approaches^31,32,42,45,46^ The vast majority of these M^pro^ inhibitors employ covalent targeting of the catalytic cysteine, with several promising noncovalent inhibitors with low nanomolar potency also entering into development. Recently, an M^pro^ inhibitor developed by Pfizer (PF-07321332) received emergency authorization, thus becoming the first M^pro^ inhibitor in clinical use^41^. However, none of these inhibitors have been developed by a process that takes into account drug resistance.

In this study we have taken decisive steps towards this goal and defined the substrate envelope of SARS-CoV-2 M^pro^. The high quality of structural data, together with our comprehensive analysis allowed us to identify the conserved features required for recognizing the diverse substrate sequences and to define the substrate envelope. We revealed that specific interactions with protease active site residues as well as a conserved network of water molecules ensure substrate specificity and proper geometry. The C-terminal side of the substrates were more divergent in their binding modes and had weaker inter-molecular interactions, consistent with the reaction mechanism. In addition to molecular recognition of substrates, the cocrystal structures enabled defining the viral substrate envelope. The conserved substrate envelope defines the molecular interactions that underlie the requirement for proper processing of the viral polypeptides, and thereby imposes an evolutionary constraint on the survival of the virus. As such, the virus cannot evolve mutations that disrupt these conserved interactions without compromising viral survival.

We have also analyzed how four M^pro^ inhibitors, including the compound in clinical use, fit into the substrate envelope. Our analysis revealed that many of the most promising inhibitors have suboptimal binding profiles that engage residues beyond the substrate envelope, most notably Met49, Asn142, Met165, Glu166 and Gln189. This suggests that SARS-CoV-2 could develop resistance to these agents by mutating at these sites, as changes to these residues would affect inhibitor binding without affecting substrate interactions or activity. Our predictions are supported by our recent comprehensive saturation mutagenesis analysis^34^ and by natural variation within M^pro^ from other coronaviral species.

As the COVID-19 pandemic continues into a third year, second and third generation SARS-CoV-2 antivirals are necessary both to thwart the rapid evolution of variants of concern and to prepare for future outbreaks. Development of additional M^pro^ inhibitors is, therefore, of critical importance as antiviral drugs targeting the proteolytic activity of M^pro^ are proven effective. In this context, using substrate envelope we determined here will enable incorporation of drug resistance considerations into the M^pro^ inhibitor development pipeline. While the prevalence of clinical resistance may depend on various factors, constraining inhibitors within the substrate envelope to leverage conserved biological features is a powerful strategy to curb evolution and prolong the longevity of the next generation M^pro^ inhibitors.

## Methods

### Expression and purification of SARS-CoV-2 M^pro^

His_6_-SUMO-SARS-CoV-2 M^pro^(C145A) was cloned into a pETite vector. Hi-Control BL21(DE3) *E. coli* cells were then transformed with this vector using standard techniques. A single colony was used to start an overnight culture in LB + kanamycin media. This culture was used to inoculate 2 × 1 L cultures in TB, supplemented with 50 mM sodium phosphate pH 7.0 and 50 µg/mL kanamycin. These cultures grew in Fernbach flasks at 37 °C while shaking at 225 rpm, until the OD600 reached approximately 1.5, at which point the temperature was reduced to 19 °C and 0.5 mM IPTG (final) was added to each culture. The cells were allowed to grow overnight. The cell mass was then resuspended in IMAC_A buffer (50 mM Tris pH 8.0, 400 mM NaCl, 1 mM TCEP) prior to lysis by three passes through a cell homogenizer at 18,000 psi. Cell lysate was then clarified by centrifugation at 45,000 × *g* for 30 min. Clarified lysate was flowed through a 5 mL Ni-Sepharose excel column on an AKTA FPLC. The column was pre-equilibrated with 5 CV of IMAC_A. The material was flowed using a sample pump with a flow rate of 5 mL/min. Following column loading, the column was washed with IMAC_A buffer until the A280 stabilized, at which point it was reset to 0. The material was then slowly eluted with a linear gradient of IMAC_B (50 mM Tris pH 8.0, 400 mM NaCl, 500 mM imidazole, 1 mM TCEP) over 40 column volumes. The presence of M^pro^ in the elution peak was confirmed by ESI-LC/MS. The SUMO tag was then cleaved by addition of ULP1 to the pooled fractions from the IMAC purification, resulting in an authentic N-terminus. Cleavage proceeded at room temperature overnight while dialyzing into 3 L of IMAC_A Buffer via 10,000 MWCO dialysis cassette. The protein was then flowed over 5 mL of Ni-NTA resin pre-equilibrated with IMAC_A buffer to remove the cleaved tag. The remaining protein was “pushed” out of the resin with an additional 5 mL wash with IMAC_A buffer. Protein was concentrated to approximately 3 mL prior to purification via SEC. A Superdex 75 16/60 column was pre-equilibrated with fresh SEC Buffer and the protein was flowed through the column at 1 mL/min while collecting 1.5 mL fractions. Fractions in the included peak were pooled and concentrated, then stored at -80 °C. We noticed that during purification the protein behaved better if kept at room temperature. Exposure for long periods of time to lower temperatures (e.g.: a cold room) mostly led to precipitation.

### Protein crystallization

Purified SARS-CoV2-M^pro^, in the inactive form (C145A), and lyophilized substrate peptides were provided by Novartis Institutes for Biomedical Research. M^pro^-NSP substrate and product cocrystals were produced according to conditions previously described by our group^47,48^ with some modifications. 10 mg/mL of protein was thawed on ice and diluted to 6.7 mg/mL in 20 mM HEPES pH 7.5, 300 mM NaCl buffer. Prior to complex formation, the protein was centrifuged at 13,000 *xg* for 1 min at 4 °C to remove insoluble particulates that may hinder crystal growth. Substrate and product complexes were formed by incubating M^pro^ with 10-fold molar excess of substrate peptides on ice for 1 h. Crystals were grown using 24-well, pre-greased, VDX hanging-drop trays (Hampton Research Corporation) at various protein to precipitant ratios (1 μL:2 μL, 2 μL:2 μL, and 3 μL:2 μL) with 10–20% (w/v) PEG 3350, 0.20–0.30 M NaCl, and 0.1 M Bis-Tris-Methane:HCl pH 5.5. Crystal growth took place at room temperature and required 1–2 weeks to obtain diffraction quality cocrystals. In some cases, crystal growth greatly benefited from micro-seeding. To limit vibration, crystallization trays were placed on foam padding.

### Data collection and structure determination

X-ray diffraction data was collected at 100 K. Cocrystals were soaked in cryogenic solutions made by supplementing the exact precipitant solutions with 25% glycerol except for the M^pro^ substrate structure C145A-NSP 7/8 where 20% ethylene glycol was used. Crystallographic data was collected locally at the University of Massachusetts Chan Medical School X-Ray Crystallography Core facility and at the Brookhaven National Laboratory NSLS-II Beamline 17-ID-2 (FMX). In-house data collection was performed with a Rigaku MicroMax-007HF x-ray generator with either a Saturn944 or HyPix-6000HE detector. Diffraction data was indexed, integrated, and scaled using HKL2000 (HKL Research Inc.) or CrysAlis^pro^PX (Rigaku Corporation). NSLS-II collected diffraction intensities were automatically indexed, integrated, and scaled using XDS^49^. All structures were determined using molecular replacement with PHASER^50^. Model building and refinement were performed using Coot^51^ and Phenix^52^. The reference model used was PDB 7L0D [https://www.rcsb.org/structure/7L0D]^47^. Prior to molecular replacement, the model was modified by removing all water, buffer, and cryogenic molecules as well as the small molecule inhibitor in the active site. To minimize reference model bias, 5% of the data was reserved to calculate R_free_^53^. X-ray data collection parameters and refinement statistics are presented in Tables 1 and 2 in the supplementary data section. Omit maps for ligands are presented in Supplementary Fig. 7.

### Structural analysis: hydrogen bonds, van der Waals calculations and the substrate envelope

The co-crystal structures contained either a M^pro^ monomer or dimer in the asymmetric unit. For complexes with a dimer in the asymmetric unit, the protease chain with better electron density around the active site and substrate was chosen for analysis. The chain D was chosen for nsp4-nsp5, nsp5-nsp6, nsp8-nsp9, nsp9-nsp10; and chain C for nsp10-nsp11, nsp15-nsp16. Hydrogen bonds were determined using the show_contacts PyMOL Plugin with default parameters where the bond angle is between 63 and 180 degrees and the distance less than 4.0 A for any and 3.6 A for an ideal hydrogen bond between the proton and heavy atom.

Prior to van der Waals calculations, the crystal structures were prepared using the Schrodinger Protein Preparation Wizard^54^. Hydrogen atoms were added, protonation states determined, and the hydrogen bonding network was optimized. A restrained minimization was performed using the OPLS2005 force field^55^ within an RMSD of 0.3 Å. All crystallographic waters were retained during structure minimization. Interaction energies between the inhibitor and protease were estimated using a simplified Lennard-Jones potential, as previously described^56^.

To generate the substrate envelope^57^ and other analyses, the cocrystal structures were superimposed using the carbon alpha atoms of active site residues 41, 144, 145, 163, 164 within one monomer. After superimposition, a Gaussian object map was generated for each substrate where the van der Waals volume was mapped onto a grid with a spacing of 0.5 Å. The intersecting volumes of 4 substrates for all 126 possible combinations of the 9 substrates were calculated. Summation of these maps generated the consensus volume occupied by at least 4 of the substrates, which was used to construct the substrate envelope in PyMOL. A similar method was used to generate the product envelope, where the consensus of at least 4 products out of the 5 product cocrystal structures were determined.

As a second method to generate the substrate envelope, a custom python script was written to place a 3D grid with a spacing of 0.2 Å at the active site and occupancy of each grid cell counted in the 9 cocrystal structures. The grid cell was occupied when the van der Waals volume of a substrate atom was within the cell. The grid cells were given scores between 0 and 1 by normalizing the occupancy by the number of structures, and the resulting substrate envelope was visualized by coloring according to the calculated scores. The figures were generated using Matplotlib^58^, PyMOL and Maestro by Schrödinger LLC.

### Reporting summary

Further information on research design is available in the Nature Research Reporting Summary linked to this article.

## Supplementary information

Supplementary Information

Reporting Summary

## Data Availability

The data that support this study are available from the corresponding authors upon reasonable request. The crystal structures determined in the current study are available in the Protein Data Bank (https://www.rcsb.org) with accession codes 7T70, 7T8M, 7T8R, 7T9Y, 7TA4, 7TA7, 7TB2, 7TBT, 7TC4.

## Code availability

The custom code used in this study is publicly available at Zenodo under the 10.5281/zenodo.6512376.
