# Supplementary material for: Defining the substrate envelope of SARS-CoV-2 main protease to predict and avoid drug resistance
Source: Nat Commun. 2022 Jun 21;13:3556. doi: 10.1038/s41467-022-31210-w (PMC9211792; doi:10.1038/s41467-022-31210-w)
Supplement: Supplementary file 1 — Supplementary Information [file 41467_2022_31210_MOESM1_ESM.pdf]

Supplementary Information

Defining the Substrate Envelope of SARS-CoV-2 Main Protease to Predict and Avoid Drug Resistance

Ala M. Shaqra[1,*], Sarah Zvornicanin[1,*], Qiu Yu Huang[1,*], Gordon J. Lockbaum[1], Mark Knapp[2], Laura Tandeske[2], David T. Barkan[2], Julia Flynn[1], Daniel N.A. Bolon[1], Stephanie Moquin[2], Dustin Dovala[2], Nese Kurt Yilmaz[1,#], Celia A. Schiffer[1,#]

[1] Department of Biochemistry and Molecular Biotechnology, University of Massachusetts Chan Medical School, Worcester, Massachusetts 01605, United States

[2] Novartis Institutes for Biomedical Research, Emeryville, CA 94608, USA

*These authors contributed equally

#These authors jointly supervised this work

Supplementary Table 1: Crystallization and refinement statistics of SARS-CoV2-M^{pro}-C145A in complex with substrate cleavage sites between nonstructural proteins (nsp).

Substrate Complexes	**nsp4 - nsp5**	**nsp5 - NSP 6**	**nsp7- nsp8**	**nsp8- nsp9**	**nsp9- nsp10**	**nsp10- nsp11**
PDB ID	7T70	7T8M	7T8R	7T9Y	7TA4	7TA7
**Data Collection**						
Location	Home source	Home source	Home source	Home source	NSLS-II	Home source
Resolution range (Å)	29.82 - 2.354 (2.438 - 2.354)	23.92 - 1.6 (1.657 - 1.6)	24.37 - 1.74 (1.802 - 1.74)	28.93 - 2.18 (2.258 - 2.18)	27.20 - 1.781 (1.845 - 1.781)	23.33 - 2.281 (2.362 - 2.281)
Space group	$P2_12_12_1$	$P2_12_12_1$	C2	$P2_12_12_1$	C2	$P12_11$
a, b, c (Å)	67.4, 99.7, 101.8	67.6, 99.7, 103.4	98.6, 80.0, 51.9	67.2, 98.9, 101.6	101.8, 78.8, 103.8	46.7, 96.4, 67.4
α, β, γ (º)	90, 90, 90	90, 90, 90	90, 114.5, 90	90, 90, 90	90, 115.5, 90	90, 103.2, 90
Total reflections	202351 (17367)	466655 (22545)	270230 (16820)	219740 (17048)	135086 (12711)	95173 (8250)
Unique reflections	28782 (2756)	85584 (7267)	37551 (3732)	35387 (3381)	69743 (6769)	26383 (2538)
Multiplicity	7.0 (6.3)	5.5 (3.1)	7.2 (4.5)	6.2 (5.0)	1.9 (1.9)	3.6 (3.2)
Completeness (%)	98.74 (96.23)	92.38 (79.46)	98.53 (99.28)	98.24 (95.64)	98.48 (96.40)	99.41 (96.14)
Average I/σ	12.96 (3.17)	22.08 (2.51)	22.39 (2.16)	14.33 (3.75)	9.46 (1.71)	9.62 (3.04)
Wilson B-factor	26.08	10.18	21.13	25.82	21	29.14
R_{merge}[a]	0.1284 (0.5863)	0.05001 (0.4511)	0.06852 (0.5145)	0.08742 (0.4391)	0.05304 (0.4258)	0.09104 (0.3705)
$CC_{1/2}$	0.997 (0.882)	0.999 (0.813)	0.999 (0.925)	0.998 (0.897)	0.998 (0.709)	0.995 (0.85)
**Refinement**						
R_{factor}[c]	0.1715 (0.2172)	0.1605 (0.2020)	0.1845 (0.2554)	0.1794 (0.2334)	0.1750 (0.2380)	0.2048 (0.2505)
R_{free}[d]	0.2199 (0.2930)	0.1971 (0.2396)	0.2278 (0.2849)	0.2363 (0.3140)	0.2163 (0.2603)	0.2545 (0.3131)
RMSD[b] in:						
Bond lengths (Å)	0.002	0.016	0.018	0.003	0.008	0.002
Bond angles (º)	0.50	1.47	1.63	0.59	0.97	0.40
Ramachandran:						
Favored (%)	96.49	97.75	97.08	97.41	98.71	96.15
Allowed (%)	3.51	2.09	2.92	2.59	1.29	3.85
Outliers (%)	0	0.16	0	0	0	0
Rotamer outliers (%)	1.49	0.19	1.12	0.58	0.57	0.6
B-factors:						
Average	29.05	15.91	28.23	28.65	26.42	32.8
Macromolecules	27.82	14.07	26.95	28.11	24.96	32.6
Solvent	36.23	26.65	36.85	34.25	35.81	36.33

[a]$R_{sym} = \Sigma \mid I - \langle I \rangle \mid / \Sigma I$, where I = observed intensity, $\langle I \rangle$ = average intensity over symmetry equivalent.
[b]RMSD, root mean square deviation.
[c]$R_{factor} = \Sigma \mid\mid F_o\mid - \mid F_c\mid\mid / \Sigma\mid F_o\mid$.
[d]R_{free} was calculated from 5% of reflections, chosen randomly, which were omitted from the refinement process.
Statistics for the highest-resolution shell are shown in parentheses.

Supplementary Table 1 continued.

Substrate Complexes	**nsp12- nsp13**	**nsp13 - nsp14**	**nsp15- nsp16**
PDB ID	7TB2	7TBT	7TC4
**Data Collection**			
Location	Home source	Home source	Home source
Resolution range (Å)	24.37 - 1.8 (1.864 - 1.8)	22.86 - 2.452 (2.539 - 2.452)	25.77 - 1.94 (2.009 - 1.94)
Space group	C2	C2	$P12_11$
a, b, c (Å)	99.0, 79.4, 51.9	98.0, 78.0, 51.8	54.8, 102.8, 67.6
α, β, γ (º)	90, 114.66, 90	90, 114.40, 90	90, 92.59, 90
Total reflections	67722 (6722)	36712 (3394)	262729 (17543)
Unique reflections	33884 (3375)	12692 (1226)	55231 (5476)
Multiplicity	2.0 (2.0)	2.9 (2.8)	4.8 (3.2)
Completeness (%)	98.90 (99.14)	95.40 (94.94)	99.83 (99.82)
Average I/σ	19.45 (3.36)	9.58 (3.89)	12.74 (1.53)
Wilson B-factor	20.08	34.97	18.41
R_{merge}[a]	0.02335 (0.1794)	0.06682 (0.2865)	0.09075 (0.5802)
$CC_{1/2}$	0.999 (0.942)	0.995 (0.939)	0.995 (0.671)
**Refinement**			
R_{factor}[c]	0.1876 (0.2495)	0.2102 (0.2831)	0.1821 (0.2427)
R_{free}[d]	0.2179 (0.2973)	0.2664 (0.3582)	0.2267 (0.2941)
RMSD[b] in:			
Bond lengths (Å)	0.011	0.002	0.01
Bond angles (º)	1.11	0.52	1.03
Ramachandran:			
Favored (%)	98.38	97.11	97.58
Allowed (%)	1.62	2.89	2.26
Outliers (%)	0	0	0.16
Rotamer outliers (%)	0	3.08	0.75
B-factors:			
Average	23.96	38.95	23.29
Macromolecules	22.73	38.89	22.41
Solvent	33.07	40.02	30.59

[a]$R_{sym} = \Sigma \mid I - \langle I \rangle \mid / \Sigma I$, where I = observed intensity, $\langle I \rangle$ = average intensity over symmetry equivalent.
[b]RMSD, root mean square deviation.
[c]$R_{factor} = \Sigma \mid\mid F_o \mid - \mid F_c \mid\mid / \Sigma \mid F_o \mid$.
[d]R_{free} was calculated from 5% of reflections, chosen randomly, which were omitted from the refinement process.
Statistics for the highest-resolution shell are shown in parentheses.

Supplementary Table 2: Crystallization and refinement statistics of SARS-CoV2-M^{pro}-C145A in complex with proteolyzed cleavage sites between nonstructural proteins (nsp).

Product Complexes	**nsp 4**	**nsp 5**	**nsp 6**	**nsp 7**	**nsp 8**	**nsp 10**
PDB ID	7MB4	7MB5	7MB6	7MB7	7MB8	7MB9
**Data Collection**						
Location	NSLS-II	NSLS-II	NSLS-II	NSLS-II	NSLS-II	NSLS-II
Resolution range (Å)	29.6 - 1.83 (1.895 - 1.83)	28.76 - 1.6 (1.657 - 1.6)	28.31 - 2.211 (2.29 - 2.211)	29.09 - 2.02 (2.092 - 2.02)	29.49 - 1.624 (1.682 - 1.624)	29.24 - 1.814 (1.879 - 1.814)
Space group	$P12_11$	$P2_12_12_1$	C2	C2	$P12_11$	$P12_11$
a, b, c (Å)	69.7, 100.6, 99.3	67.9, 100.1, 103.1	124.9, 80.8, 62.7	99.4, 79.4, 52.1	67.8, 103.0, 104.3	54.2, 97.9, 67.5
α, β, γ (º)	90, 104.2, 90	90, 90, 90	90, 91.3, 90	90, 114.5, 90	90, 101.4, 90	90, 102.5, 90
Total reflections	230216 (22187)	180287 (17298)	61772 (5885)	47317 (4338)	344159 (28355)	120841 (10414)
Unique reflections	115488 (11277)	90251 (8704)	31114 (3000)	23874 (2236)	174513 (15578)	61128 (5401)
Multiplicity	2.0 (2.0)	2.0 (2.0)	2.0 (2.0)	2.0 (1.9)	2.0 (1.8)	2.0 (1.9)
Completeness (%)	98.70 (97.32)	96.72 (94.07)	99.35 (95.45)	98.30 (93.04)	98.66 (88.38)	98.21 (86.91)
Average I/σ	12.51 (2.46)	12.65 (2.34)	12.72 (1.89)	10.18 (1.90)	11.69 (1.75)	10.12 (1.44)
Wilson B-factor	24.55	18.18	44.29	39.68	17.49	30.37
R_{merge}^a	0.02846 (0.2512)	0.02716 (0.2411)	0.03287 (0.3806)	0.03295 (0.2949)	0.0347 (0.375)	0.03511 (0.3266)
$CC_{1/2}$	0.999 (0.871)	0.999 (0.844)	0.999 (0.719)	0.998 (0.882)	0.999 (0.813)	0.998 (0.839)
**Refinement**						
R_{factor}^c	0.1770 (0.2443)	0.1643 (0.2333)	0.2226 (0.3122)	0.1900 (0.3084)	0.1686 (0.2506)	0.1989 (0.3138)
R_{free}^d	0.2198 (0.2730)	0.1838 (0.2500)	0.2720 (0.3951)	0.2261 (0.3490)	0.1951 (0.2853)	0.2276 (0.3457)
$RMSD^b$ in:						
Bond lengths (Å)	0.019	0.009	0.003	0.003	0.013	0.013
Bond angles (º)	1.4	1.18	0.62	0.64	1.08	1.03
Ramachandran:						
Favored (%)	97.31	98.21	95.91	97.37	98.45	96.56
Allowed (%)	2.69	1.63	4.09	2.63	1.47	3.27
Outliers (%)	0	0.16	0	0	0.08	0.16
Rotamer outliers (%)	0	0.39	0.21	0.39	0.19	0
B-factors:						
Average	30.10	22.80	53.41	50.09	23.91	36.47
Macromolecules	29.22	21.26	53.5	50.01	21.94	36
Solvent	37.73	33.87	49.8	51.49	35.52	41.74

$^aR_{sym} = \Sigma \mid I - \langle I\rangle \mid / \Sigma I$, where I = observed intensity, $\langle I\rangle$ = average intensity over symmetry equivalent.
^{b}RMSD, root mean square deviation.
$^cR_{factor} = \Sigma \mid\mid F_o\mid - \mid F_c\mid\mid / \Sigma\mid F_o\mid$.
$^dR_{free}$ was calculated from 5% of reflections, chosen randomly, which were omitted from the refinement process.
Statistics for the highest-resolution shell are shown in parentheses

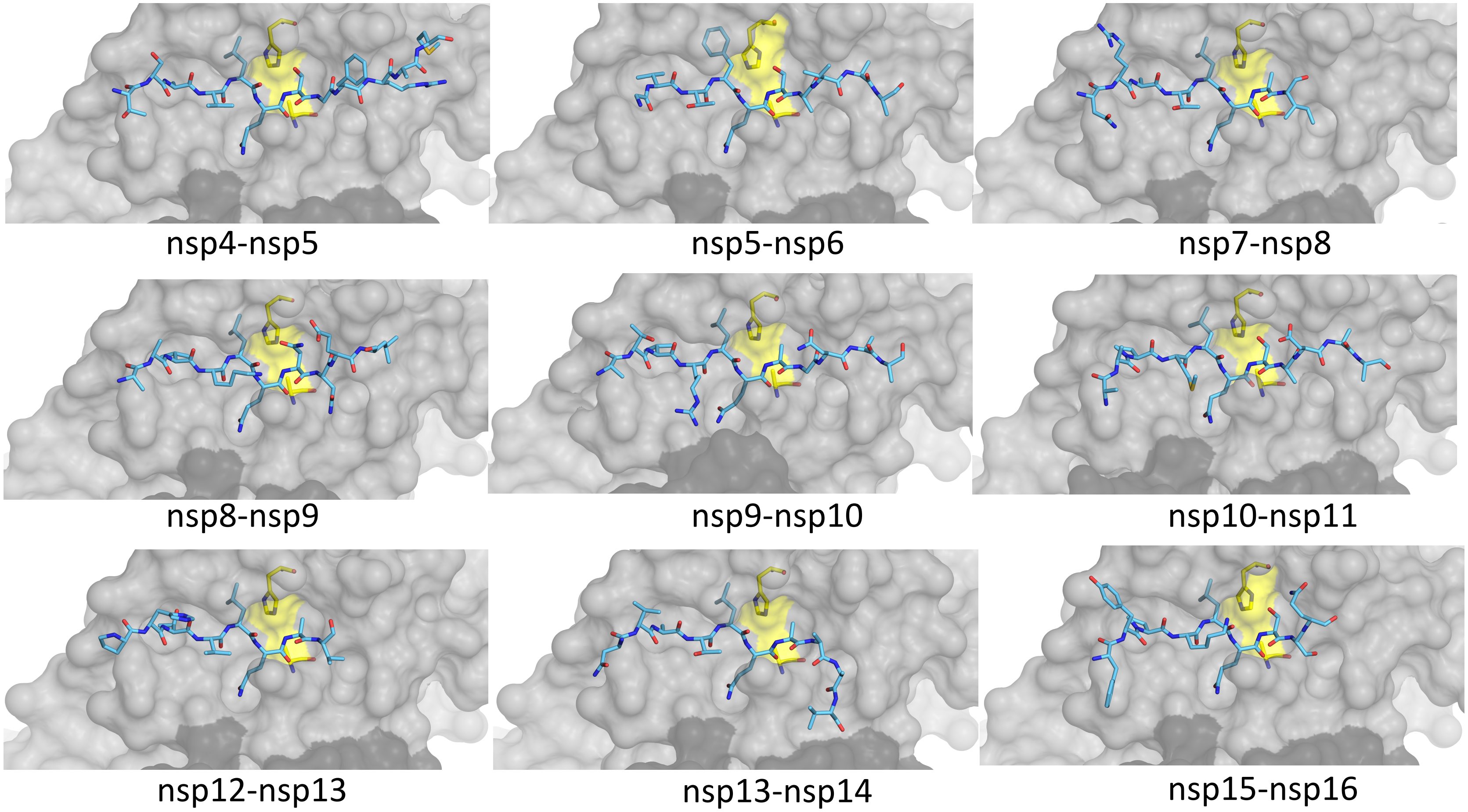

Supplementary Figure 1. Substrates bound at the active site of M^{pro} in the cocrystal structures determined. The peptide is depicted as cyan sticks and the catalytic dyad is colored yellow. The panels show close-up view of the active sites, with the protease in gray surface representation, similar to Figure 1C of the main manuscript.

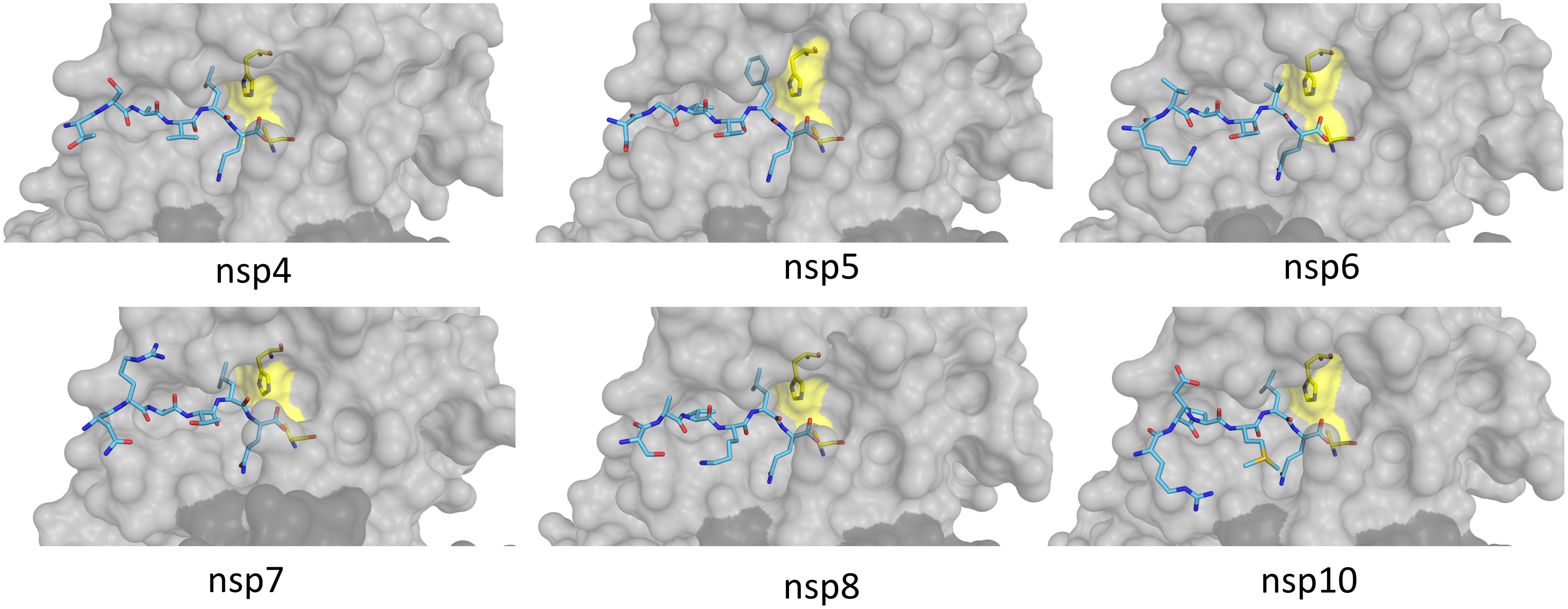

Supplementary Figure 2. Substrates cleaved with the N-terminal product bound at the active site of M^{pro} in the cocrystal structures determined. The peptide is depicted as cyan sticks and the catalytic dyad is colored yellow. The panels show close-up view of the active sites, with the protease in gray surface representation.

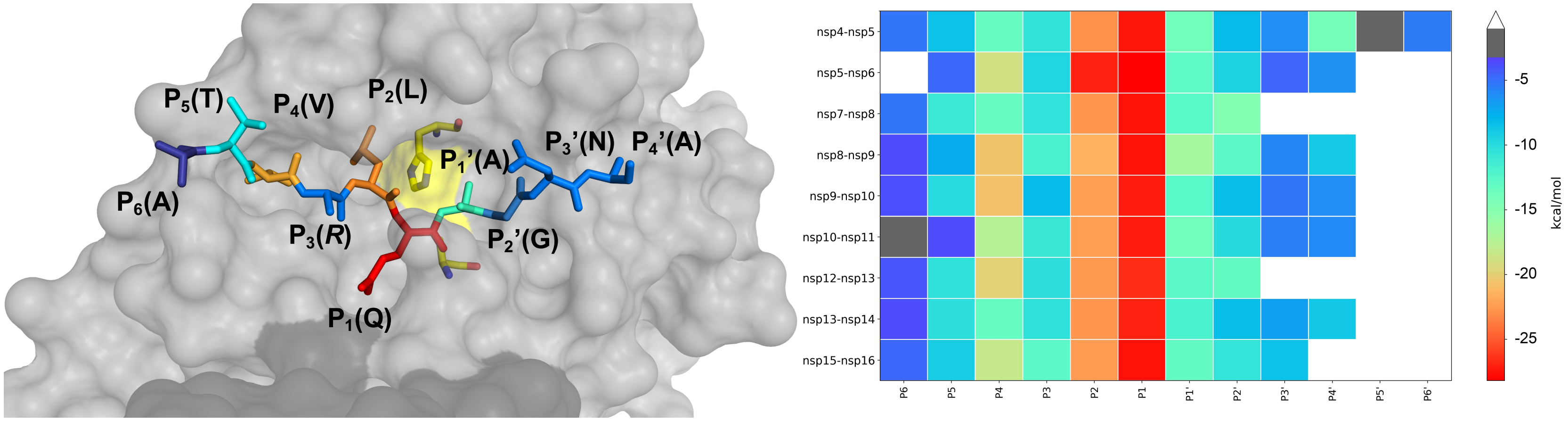

Supplementary Figure 3. Substrate vdW interactions with the active site of M^pro^ in the cocrystal structures. Similar to Figure 3A of the main manuscript, the peptide is depicted as sticks and the catalytic dyad is colored yellow. The substrate residues are colored from blue to red according to increasing van der Waals interactions with the protease. The heat map shows per residue interactions for all 9 substrate cocrystal structures.

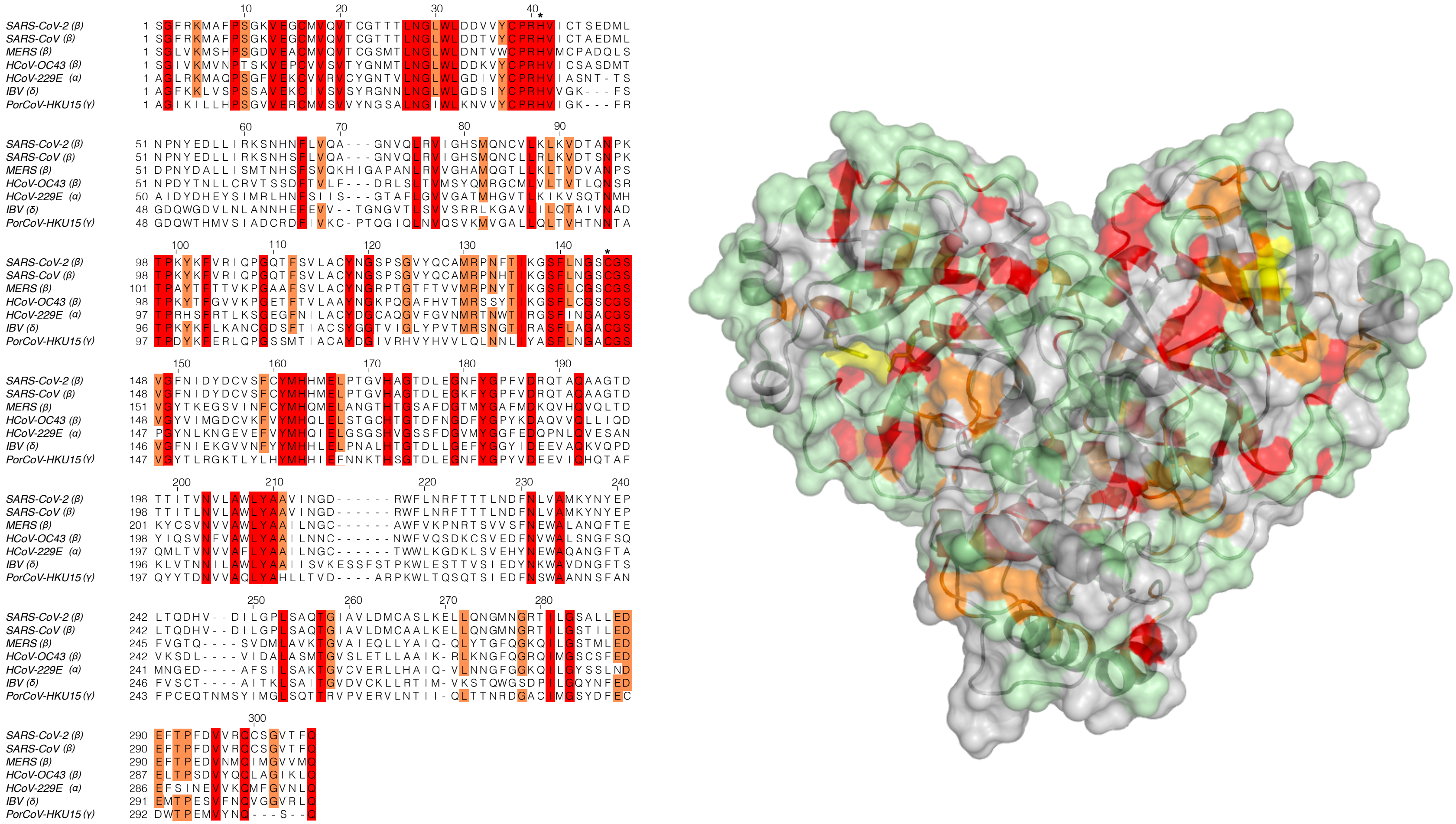

Supplementary Figure 4. Sequence conservation of M^{pro}. Alignment of 7 diverse sequences coronaviral sequences of M^{pro} -- Beta: SARS-CoV-2, SARS-CoV, MERS, Human coronavirus OC43 (HCoV-OC43); alpha: Human coronavirus 229E (HCoV-229E); delta: Avian coronavirus IBV; gamma: Porcine Coronavirus HKU15(PorCoV-HKU15) (left) and the amino acid sequence conservation of M^{pro} betweenthe 7 coronaviral species depicted on the structure where surface residues identical are shown in all 7 (red), 5-6 of 7 (orange), 3-4 of 7 (green) and less than 3 (highly variable; gray) sequences are indicated by color (right).

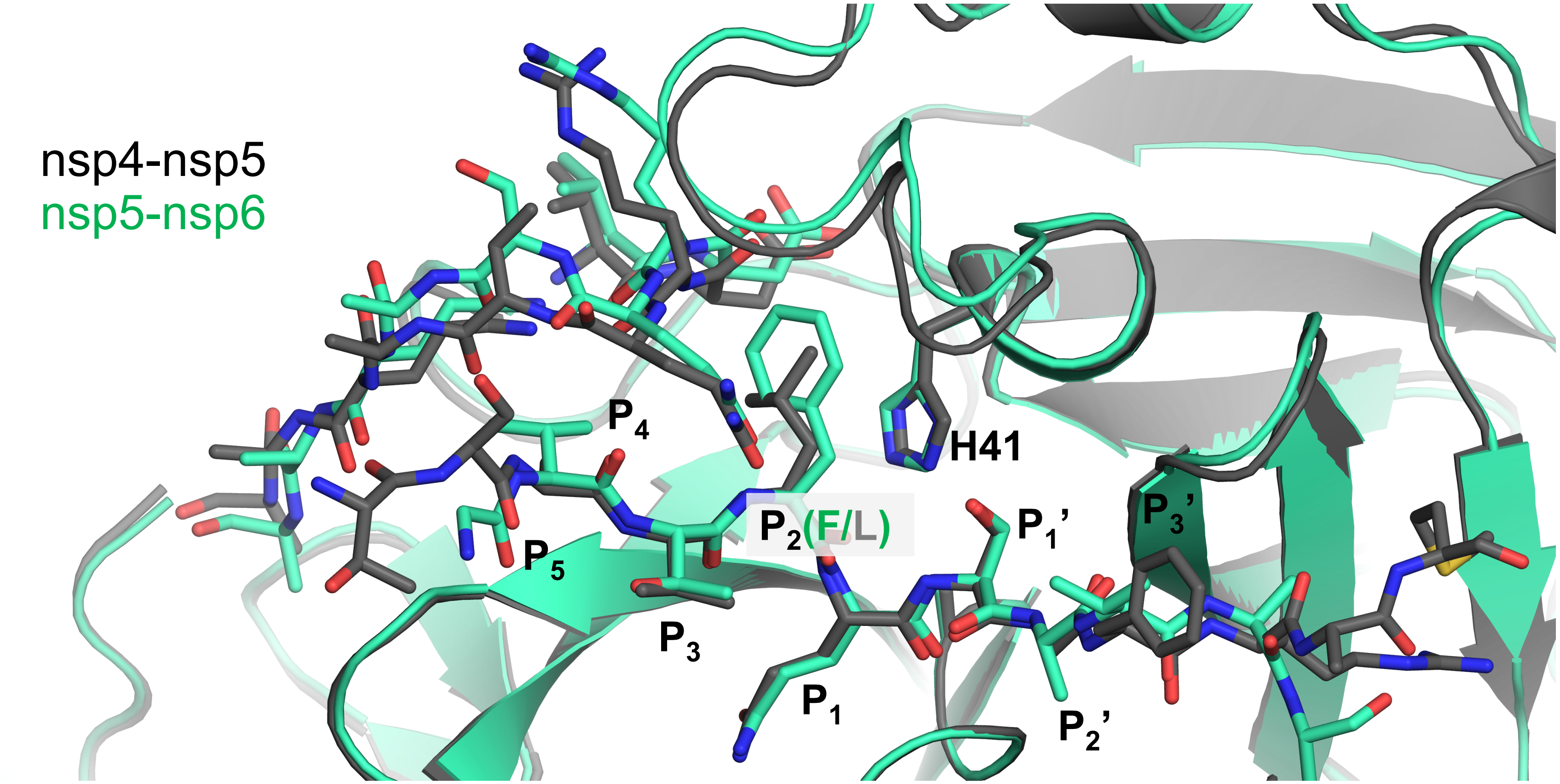

Supplementary Figure 5. Comparison of the variable 187-192 loop in substrate–M^pro cocrystal structures. The substrate and the loop residues depicted as sticks as well as the catalytic His side chain. Nsp5-nsp6 with a Phe at P2 is the most divergent (in green), shown in comparison with nsp4-nsp5.

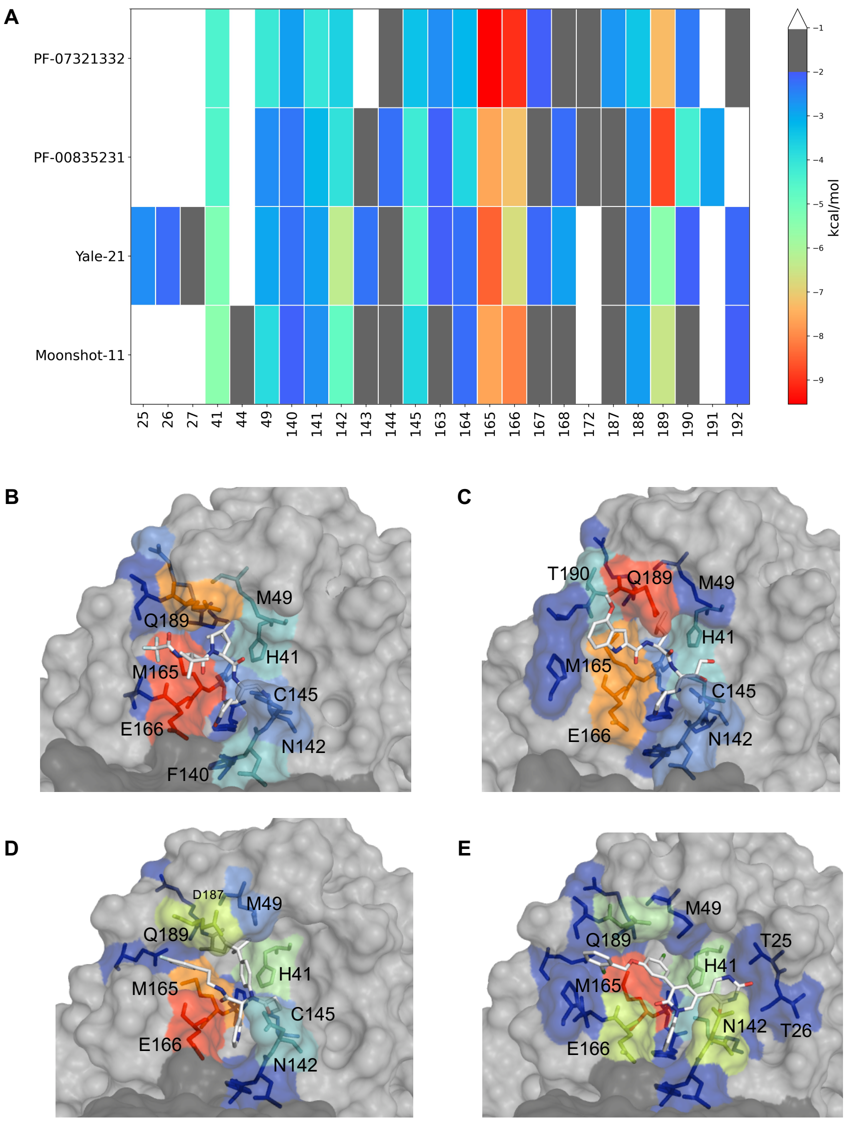

Supplementary Figure 6. Inhibitor van der Waals interactions on the active site of M^{pro} **(A)** colored by extent of van der Waals interactions for **(B)** PF-07321332 (35), **(C)** PF-00835231 (36), PDBID: 6XHM **(D)** Noncovalent potent compound 21 (37), PDBID: 7L13 **(E)** Moonshot compound 11 (38), PDBID: 7NW2.

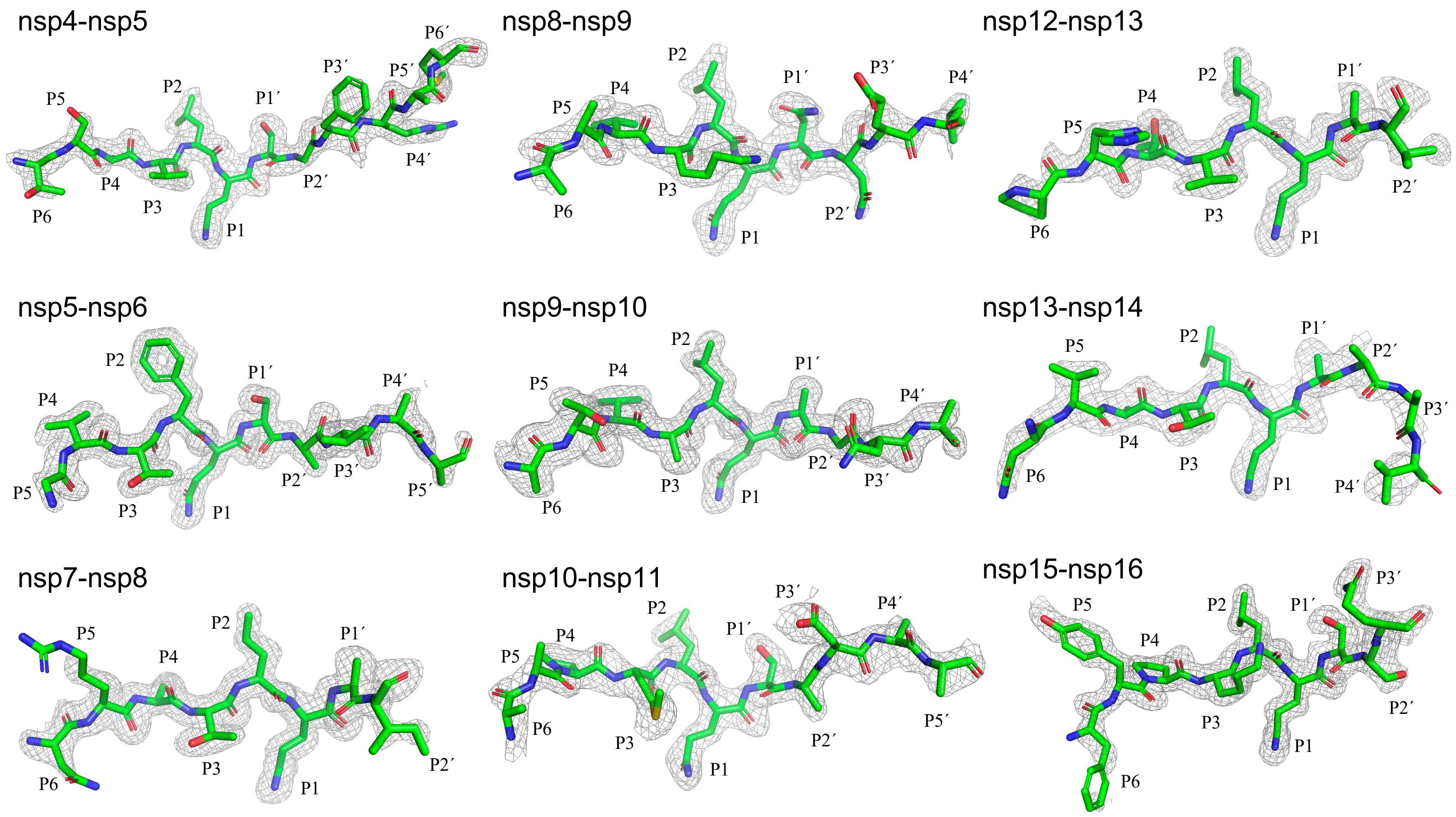

Supplementary Figure 7. Omit maps for the bound ligand in substrate–M^pro cocrystal structures. The substrate peptide is depicted as green sticks inside the electron density.
